# What determines positive, neutral, and negative impacts of *Solidago canadensis* invasion on native plant species richness?

**DOI:** 10.1038/srep16804

**Published:** 2015-11-17

**Authors:** Li-Jia Dong, Hong-Wei Yu, Wei-Ming He

**Affiliations:** 1 State Key Laboratory of Vegetation and Environmental Change, Institute of Botany, Chinese Academy of Sciences, Haidian District, Beijing 100093, China

## Abstract

Whether plant invasions pose a great threat to native plant diversity is still hotly debated due to conflicting findings. More importantly, we know little about the mechanisms of invasion impacts on native plant richness. We examined how *Solidago canadensis* invasion influenced native plants using data from 291 pairs of invaded and uninvaded plots covering an entire invaded range, and quantified the relative contributions of climate, recipient communities, and *S. canadensis* to invasion impacts. There were three types of invasion consequences for native plant species richness (i.e., positive, neutral, and negative impacts). Overall, the relative contributions of recipient communities, *S. canadensis* and climate to invasion impacts were 71.39%, 21.46% and 7.15%, respectively; furthermore, the roles of recipient communities, *S. canadensis* and climate were largely ascribed to plant diversity, density and cover, and precipitation. In terms of direct effects, invasion impacts were negatively linked to temperature and native plant communities, and positively to precipitation and soil microbes. Soil microbes were crucial in the network of indirect effects on invasion impacts. These findings suggest that the characteristics of recipient communities are the most important determinants of invasion impacts and that invasion impacts may be a continuum across an entire invaded range.

There is a continuing debate on whether plant invasions pose a great threat to native plant diversity (i.e. invasion paradox)^1^,^2^,^3^,^4^,^5^. Field studies show that plant invasions can dramatically reduce native plant species richness or diversity^6^,^7^,^8^,^9^,^10^. Correlation analyses demonstrate there is a positive relationship between invasive plant dominance and native plant decline^11^,^12^. Meta-analyses suggest that invasive plants tend to pose harmful effects on native plant species^2^,^13^,^14^. Accordingly, invasive plants are widely accepted as one of the leading direct causes of species loss.

However, increasing evidence has suggested that the impacts of invasive species on native biodiversity may not be so dire. For example, in some regions of the world, plant invasions exceed extirpations thereby increasing species richness^15^,^16^ or plant invasions increase native species richness through facilitation in some cases^2^,^17^,^18^. Some field studies at fine scales demonstrate that plant invasions have no significant impacts on species richness (i.e. neutral)^2^,^19^,^20^,^21^. Additionally, there is a positive correlation between native species decline and invasive species dominance, but this correlation does not necessarily mean that invasive species are the determinants of the observed change^11^.

This invasion paradox can be ascribed to diverse causes. First, the crux of the paradox concerns positive associations between native and exotic species richness at broad spatial scales, and negative associations at fine scales^1^,^3^,^4^. In other words, the assessment of invasion impacts on native species richness is strongly influenced by space scales^3^,^13^,^14^,^18^,^22^. Second, the impacts of invasive plants on native diversity change dramatically over time^18^,^23^,^24^. Third, the impacts of invasive species are dependent not only on invader attributes but also on characteristics of the invaded community^25^,^26^. Finally, the discrepancies among different studies may be implicated to different research methods, like observational versus experimental field studies.

Despite this continuing debate, there is little information in the literature for what determines contrasting invasion impacts on diversity, with an exception of space-dependence^3^,^14^,^18^,^22^. Invasion impacts on native diversity may depend on space scales, but this dependence is not universal^4^. Climate, recipient communities, and invaders are the most crucial determinants of invasion impacts. For example, climate can determine the invaded range of invasive plants^27^, the characteristics of a recipient community govern its invasibility^25^,^28^, and the traits of invasive plants shape their invasiveness^26^,^29^. Obviously, these determinants control the invasion impacts as a whole. No studies, however, have explicitly examined the relative contribution of climate, recipient communities and invaders to invasion impacts in the context of multiple dimensions.

Assessing invasion impacts on native plants at small scales is highly necessary for biological control and conservation^30^. Most studies on the impact of a single invasive species have been limited to single or a few study locations^31^, thereby not shaping a whole picture of invasion impacts across an entire invaded range. For a given invader, increasing study locations are required to better understand invasion impacts and the related mechanisms^25^,^26^,^31^.

We chose *Solidago canadensis* as our focal invader based on the following reasons: it is a worldwide invasive plant and invades diverse communities or habitats^32^, and it is assumed to pose negative effects on native diversity^33^. Species richness has been proven to be a relevant indicator to determine the biodiversity of a given area, as well as a valuable element to be used by decision makers at different levels to establish spatially explicit conservation strategies for biodiversity^17^,^34^,^35^. Accordingly, to examine the impacts of *S. canadensis* invasion on native plant species richness across an entire invaded range and elucidate the associated mechanisms, we conducted a comparative field study based on 291 pairs of invaded and uninvaded plots. Specifically, we addressed the following questions: (1) whether the impacts of *S. canadensis* invasion on native plant species richness are unidirectional or not across the invaded range? and (2) how climate, recipient community characteristics and *S. canadensis* traits differentially determine invasion impacts on native plant species richness?

## Methods

### Study species

*Solidago canadensis* L. is native to North America and was intentionally introduced into China as an ornamental plant in 1913^36^. This invasive species has a broad range climatic adaption and can grow under a wide range of soil conditions in its native habitats^37^. *Solidago canadensis* grows mainly along roadsides, abandoned agricultural lands and other disturbed habitats. Presently this invasive plant can be found widely distributed in several provinces of eastern China, such as Jiangsu, Shanghai, Zhejiang, Jiangxi, and Anhui^36^.

### Field survey and sample analyses

**Figure 1:**
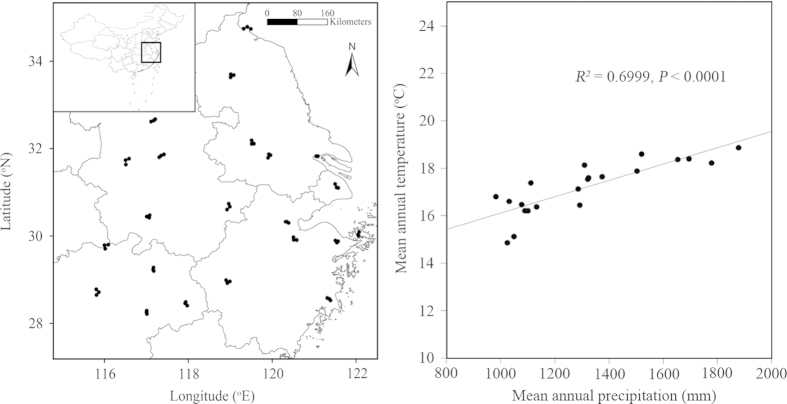
Twenty-two sampling locations and 66 sampling sites for the field survey across the invaded range by *Solidago canadensis* in China (**A**), and the relationship between mean annual precipitation and mean annual temperature for all the sampling sites from 2003 to 2013 (**B**). Meteorological data around the sites were obtained from the official website of the National Meteorological Center of China (http://www.nmc.gov.cn). The panel A (left) was generated using software ArcGIS 10.2 and the panel B (right) using software R 3.1.3.

To quantify the invasion impacts of *S. canadensis* upon the native plant species richness across its entire invaded range, we selected 22 sampling locations, each with three sites (Fig. 1A; see Table S1 for details). Accordingly, 66 sampling sites were chosen in our study (Fig. 1A). Note that *S. canadensis* was the only one invasive plant species in these sites, which was ideal for us to run the cause-effect analyses. Fieldwork was carried out from July to September 2014. We compared heavily invaded and uninvaded plots at each sampling site. We defined heavily *S. canadensis*-invaded plots as having high *S. canadensis* cover (average of 85%). Thus, each pair consisted of heavily invaded and nearby uninvaded plots. The uninvaded plot was selected so as to have as similar site conditions as possible to the invaded plots. Specifically, we surveyed five or three 1 × 1 m invaded plots and corresponding uninvaded plots. In most cases, five pairs of plots were available; in other cases, three pairs of plots were available. In total, 291 pairs of invaded and uninvaded plots were surveyed and sampled. We recorded total plant species, and their cover, density and height in all plots.

Soil samples were collected from all of the invaded and uninvaded plots. In each plot, five soil samples were taken (ca. 10 cm deep in soil) by a cylinder and then pooled as a soil sample. Each of the soil samples was divided into two portions: one for determining soil microorganisms by phospholipid fatty acid (PLFA) analysis^38^ and the other for measuring soil abiotic properties. Specifically, the fatty acids chosen to represent fungi were 18:2ω6,9c and bacteria were i14:0, 14:0, i15:0, a15:0, 15:0, a16:0, i16:0, 16:0, 16:1ω7c, 16:1ω9c, i17:0, a17:0, 17:0, cyl7:0, 18:0, 18:1ω5c, 18:1ω7c, and cyl9:0; the fungi:bacteria ratio was expressed as the ratio of 18:2ω6,9c to bacterial PLFAs^38^. pH was determined in a soil solution rate of 1:2.5 (soil:distilled water) using a pH meter (Sartorius PB-10 meter), organic carbon (C) using the potassium dichromate oxidation method, total nitrogen (N) content using Kjeldahl apparatus (FOSS 2200), and soil texture using laser particle size analyzer (Mastersizer 2000).

### Data analyses

We selected the changes in native plant species as a response variable and the others as exploratory variables. The relative contributions of climate, recipient communities, and invasive *S. canadensis* alone to invasion impacts (i.e. changes in native plant species) were quantified in light of the following data analyses. To assess the impacts of *S. canadensis* on native plant species richness, we coined a relative impact index (*RII*) as follows:


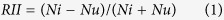


where *Ni* is the number of native plant species in each invaded plot and *Nu* is the number of native plant species in each uninvaded plot. RII has values ranging from 1 to −1, and is positive for increased native plant species due to plant invasion and negative for decreased native plant species due to plant invasion. First, we ranked RII values in descending order. Then we divided all RII values into three groups: positive, zero, and negative. Next, we calculated the means and standard errors of these groups. Finally, we tested the difference among three groups using one-way analysis of variance (SPSS 15.0, SPSS Inc., Chicago). RII was treated as a response variable in the data analyses below.

To quantify the relative contribution of fundamental determinants to invasion impacts (i.e., *RII*), we categorized these determinants into three different categories: (i) climate, (ii) recipient communities, and (iii) the invader. Specifically, climatic determinants included a mean annual temperature (MAT) and mean annual precipitation (MAP). MAT and MAP around sampling locations were obtained from the official website of the National Meteorological Center of China (http://www.nmc.gov.cn). Recipient communities included three components: native plant communities, soil microorganisms, and soil abiotic properties. The diversity of plant species in the uninvaded plots included species richness (i.e. numbers of species), Shannon-Wiener index, Simpson dominance index, and Pielou evenness index. These diversity indices were calculated as described by Magurran^39^. Soil microorganisms included bacterial PLFAs, fungal PLFAs, total PLFAs, and F/B ratio. Soil abiotic properties included soil pH, organic C, total N, and soil texture (i.e., clay [%], silt [%], and sand [%]). The traits of the invader included its cover, density and height. The above explanatory variables were presented in Table S2 and used in data analyses below.

To address how climate, recipient communities and the invader determine RII values and to quantify their direct and indirect effects, we selected the partial least squares path modeling (PLS-PM) algorithm, a technique fusing of regression analysis, principal component analysis and path analysis^40^. This approach has clear advantages over commonly used in covariance-based structural equation models, because it does not require strong assumptions with respect to the distributions of the manifest variables, the sample size or the measurement scale^40^.

The RII model was developed in a formative way. We defined MAT, MAP, native plant communities, soil microorganisms, soil abiotic properties, *S. canadensis* and RII as latent variables (LVs), an abstract concept that combined directly measured parameters. To minimize unknown effects on RII, before analysis, we firstly used stepwise regression to screen variables correlated significantly with RII for each latent variable. The finally chosen variables for each latent variable, which were directly measured (i.e., manifest variables [MVs]), were put into the path models (see Table S3 for details). Here, each latent variable was considered as a linear combination of its own manifest variables; each outer weight can be considered as a proxy for the importance of each manifest variable in the construction of the latent variable; the path coefficients were interpreted as standard regression coefficients^41^.

A bootstrapping validation technique with 200 boots was performed to evaluate the path coefficients and the weights of the manifest variables. Some studies have used the goodness of fit index (GoF) to assess the validation of the overall model^40^, but this index may be unsuitable for model validation^42^. Accordingly, we just provided the index as a reference but not as an indicator of the overall validation of the model. Only variables with significant relationships were included in the final model.

Finally, contribution of each latent variable to global explained observed variability (*R*^*2*^) of RII or *S. canadensis* was obtained through the equation:


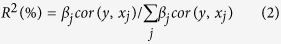


where *j* is the number of latent variables, *β* is the path coefficient estimated by bootstrapping, and *cor(y, x*_*j*_) is the correlation between explanatory variable and response variable^40^.

All statistical analyses were performed using R software. PLS-PM algorithm was performed using the package “plspm”, and the stepwise regression using the package “MASS”.

## Results

**Figure 2:**
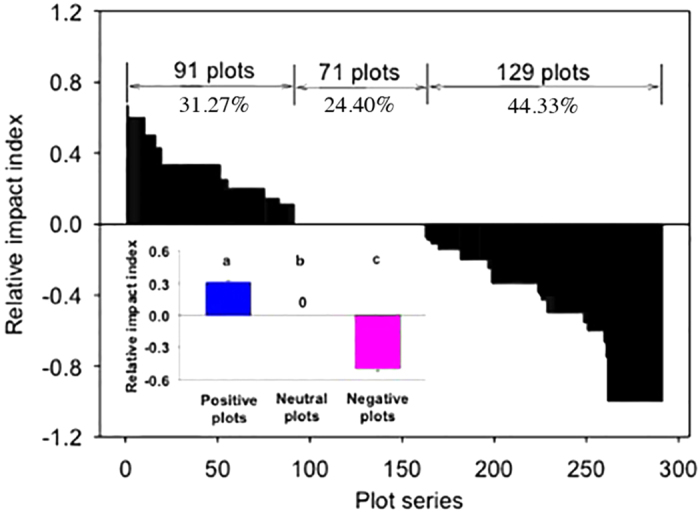
The impacts of *Solidago canadensis* invasion on native plant species richness (i.e. the number of species), as measured by relative impact index (RII) for each of the 291 pairs of invaded and uninvaded plots. We ranked 291 plots from 1 to 291 in descending RII order. The numbers above the bars show the numbers of plots with positive, neutral and negative RII values and their corresponding percents. The embedded smaller panel inside represents means (+1 SE) of RII for positive, neutral and negative plots. Different letters indicate significant differences at *P* = 0.05.

We ranked 291 plots in descending order of relative impact index (RII) (Fig. 2). There were 91 plots with positive RII values, accounting for 31.27% of all the plots; there were 71 plots in which RII values were zero, accounting for 24.40% of all the plots; there were 129 plots with negative RII values, accounting for 44.33% of all the plots (Fig. 2). Accordingly, invasive *S. canadensis* yielded three different types of impacts on native plant species richness: positive impact (RII = 0.31 ± 0.015, mean ± 1 SE), neutral impact (RII = 0) and negative impact (RII = −0.50 ± 0.028) (Small panel in Fig. 2). In other words, *S. canadensis* invasion increased native plants in some cases, decreased native plants in some cases, or had no effects on native plants in other cases.

**Figure 3:**
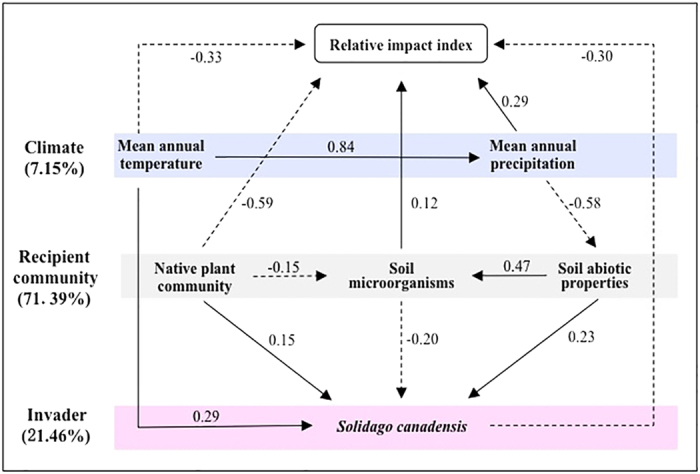
Path models examining the effects of different determinants on the relative impact index (RII) values through pathways of climate (i.e., a mean annual temperature and mean annual precipitation), recipient communities (i.e., native plant communities, soil microorganisms, and soil abiotic properties), and *Solidago canadensis* (i.e., the traits of *S. canadensis*). Solid and dash arrows indicate significantly positive and negative effects on RII (*P* < 0.05), respectively; the pathways without significant effects on RII are not shown (*P* > 0.05). Numbers associated with pathways between variables represent standardized path coefficients (scaled by the standard deviations of the variables).

The relative contributions of climate, recipient communities, and the invader to RII were 7.15%, 71.39% and 21.46%, respectively (Fig. 3; Table 1), suggesting that they differentially influenced RII. Different variables of climate, recipient communities and the invader differentially explained their contributions to RII (Tables 1 and 2).

For climatic factors, MAP contributed greater to RII than MAT (Table 1). Interestingly, MAP and MAT had the opposite direct effects on RII (Fig. 3). Across the entire invaded range, MAP was positively correlated with MAT (Fig. 1B). Accordingly, the direct effects of MAP on RII were offset by those of MAT. In terms of indirect effects, MAT largely influenced RII via MAP and *S. canadensis* and MAP mainly did RII via soil abiotic properties (Fig. 3).

**Figure 4:**
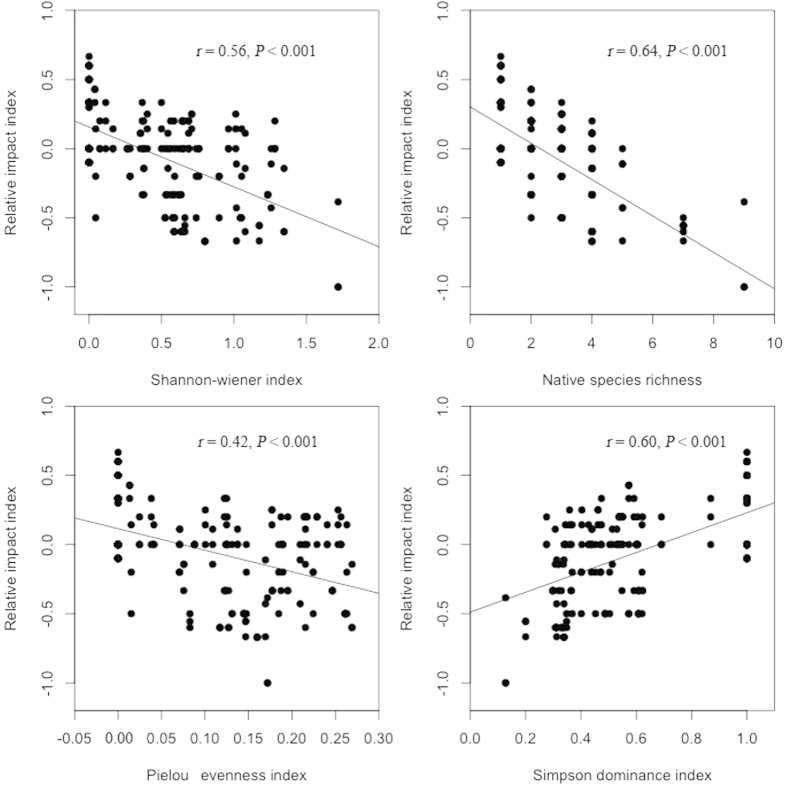
Correlations of relative impact index with Shannon-wiener index, native species richness, Pielou evenness index, and Simpson dominance index in the uninvaded plots.

For recipient communities, native plant communities had directly negative effects on RII, the opposite was the case for soil microbes, and soil abiotic properties had no direct effects on RII (Fig. 3). In terms of indirect effects, plant communities and soil abiotic properties largely affected RII through both soil microbes and *S. canadensis*, and soil microbes mainly influenced RII via *S. canadensis* (Fig. 3). The contribution of plant communities, soil microbes and soil abiotic properties to RII was 65.88%, 3.56% and 1.95%, respectively (Table 1). The contribution of plant communities was primarily explained by native species richness (Table 2), although RII was significantly correlated with Shannon-Wiener index, native species richness, Pielou evenness index, and Simpson dominance index (Fig. 4). The contribution of soil microbes was greatly ascribed to total fungi, and that of soil abiotic properties was implicated to soil texture and total N (Table 2).

Overall *S. canadensis* had directly negative effects on RII (Fig. 3). Of all the measured traits, both the cover and density of *S. canadensis* affected RII, but its height had no effect on RII (Table 2 and S3). When direct and indirect effects were considered together, climate and recipient communities explained 63.68% and 36.32% variance in the traits of *S. canadensis*, respectively (Table S4). MAT had greater effects on *S. canadensis* than MAP, and the effect of soil microbes on *S. canadensis* was roughly equal to the sum of effects of plant communities and soil abiotic properties (Table S4).

## Discussion

The first key finding of our study was that the invasive forb *S. canadensis* simultaneously had three different consequences for native plant species richness across the entire invaded range (i.e., positive, neutral, and negative impacts). In other words, invasion impacts of *S. canadensis* on native plants were variable in direction. To our knowledge, this is the first study to demonstrate that a single invasive plant species possesses three contrasting impacts on native plants based on hundreds of plots covering the entire invaded range, as positive, neutral or negative impacts were separately reported in different studies with different invasive plants^2^,^4^,^13^,^14^,^18^. If these three types of invasion impacts are universal, then they can reconcile those seemingly conflicting findings only based on single or only a few study locations. Thus it is not surprising that a single invader exhibits negative, positive or neutral impacts on native diversity thereby depending on specific locations.

We propose a few hypotheses that might explain the different invasion consequences. In our study, native plant communities, as the dominant determinant, yielded 65.88% contributions to invasion impacts. This contribution was largely ascribed to species richness and species evenness in recipient plant communities. For recipient plant communities, there might be four scenarios: high richness and high evenness, high richness and low evenness, low richness and high evenness, and low richness and low evenness. Accordingly, the initial regimes of recipient plant communities, to large extent, determine the final consequences of *S. canadensis* invasion. For instance, the impacts of *S. canadensis* invasion were smaller in those communities either with greater numbers of native species or with more species evenness, and vice versa. As for the final numbers of native plant species after *S. canadensis* invasion, decreased native plant richness may be linked to the fact that invaders can exclude most local plant species. For example, *S. canadensis* can form near monocultures after its invasion in some locations. By contrast, increased native plant richness may be linked to facilitation by invasion^43^. For example, some invaders replace the former dominant plant(s) but make habitats more suitable for other local species^2^,^17^,^44^. There is a possibility that *S. canadensis* only displaced the dominant species but had no effects on non-dominant species, thereby allowing the species richness to remain stable. Such phenomenon has been observed in previous studies, despite changes in species composition and changes in the dominant plant species^3^,^17^,^25^.

A second key finding of our study was that climate, recipient communities and *S. canadensis* differentially determined the invasion impacts on native plant species richness. In terms of the relative importance, recipient communities (71.39%) were overwhelming compared to climate (7.15%) and *S. canadensis* (21.46%). Accordingly, the characteristics of recipient communities appear to be a crucial determinant of invasion impacts. In other words, the regimes of local communities largely determine their potential to resist invasive plants. Although our work is a case study with one invader only, it provides a clear and comprehensive perception regarding the relative roles of climate, recipient communities and invaders in invasion impacts. It should be noted there is little information for how these determinants differentially influence invasion impacts and their differential contributions should be tested with a fairly wide range of invasive plants.

At the range scale, invasion impacts did not vary with latitude and longitude, and the opposite was the case for precipitation and temperature. Interestingly, precipitation and temperature, both of which were positively correlated to each other, had opposite influences on invasion impacts. This provides an indication why climate yielded a relatively low contribution to invasion impacts when precipitation and temperature as latent variables were analyzed at the same time. Additionally, we found that the indirect effect of MAT was greater than that of MAP. Accordingly, when direct and indirect effects were considered together, the net contribution of MAT to invasion impacts was smaller than that of MAP due to a counteractive effect. Teasing apart the relative roles and direct and indirect effects of precipitation and temperature could help us to understand the potential risks of plant invasions under climate change.

Despite the direct effects on invasion impacts, soil microbes were crucial to form a network of indirect effects, because soil microbes link other determinants together. For example, although soil abiotic variables had no significant direct influence on invasion impacts of *S. canadensis*, they greatly influenced invasion impacts via microbe-mediated pathways. The role of soil microbes was largely attributed to soil fungi but not soil bacteria. However, we know much less about the role of soil microbes in invasion impacts than we do about their role in invasion success. Therefore this aspect deserves further research.

We found that invasion impacts of *S. canadensis* increased with its cover, consistent with the findings by Yurkonis *et al.*^45^, who found that native species richness declined with increasing cover for three of four invaders. Additionally, the density of *S. canadensis* was positively correlated with its invasion impacts. Thus the cover and density of invasive plants, to some extent, can rapidly and effectively indicate their impacts on native plant species richness in the field.

Based on the RII values across the invaded range and their determinants, we propose a new hypothesis of an invasion impact continuum across the entire invaded range. RII values are determined by positive and negative effects so that the differences between two types of effects shape the spatiotemporal patterns of RII. For a given location in the field, its RII value depends on location-specific effects. The magnitudes of positive and negative impacts of *S. canadensis* invasions were variable, consistent with a recent meta-analysis by Ricciardi *et al.*^18^, who proposed that invasion impacts might vary in magnitude. Quantitatively, recipient communities, *S. canadensis* and climate as a whole determine the net effects of *S. canadensis* invasion through direct and indirect pathways, and the effects of determinants were either negative or positive. Thus, the complex combinations of determinants and their effects govern invasion impacts, and the net effects may be continual. Note that there are other possibilities that might explain this continuum. For example, invasion-induced herbivore shifts can influence native plant species^46^, and some invaders can exhibit competition and facilitation on native species in the long term^43^.

Note that invasion impacts may depend on spatial scales. For example, Powell *et al.*^3^ found that invasive plants exhibited scale-dependent effects on diversity by altering species-area relationships. By the same token, the impacts of *S. canadensis* invasions might vary with plot size. On the other hand, the same plot size is necessary to ensure data comparable. Taken together, it would be better to consider multiple spatial scales when addressing invasion impacts if possible, but comparative studies should be performed under the same scale.

This study has some implications for field sampling and native plant conservation. Although the importance of numerous study locations has been recognized, most impact studies have been limited to single or a few study locations^31^. Given that invasion impacts vary with space^13^,^14^,^47^,^48^, sampling locations seem to be crucial to comprehensively understand invasion impacts. If we only select a few locations, we may gain a facet of invasion impacts rather than their gamut. In this sense, invasion impacts on native plant species should be considered at the scale of the entire invaded range. *Solidago canadensis* does not always reduce native plant species richness so that retaining it *in situ* may be rational at some locations, particularly in habitats where it can increase native plant richness. Of course, context-specific assessments on relationships between invasive plants and native plants are necessary because they can help to effectively conserve native plants.

In summary, *S. canadensis* invasion might not be so dire due to the fact that about 60% of surveyed plots did not show negative impacts on native plant species richness. As Ricciardi *et al.*^18^ pointed out that invasion impacts of invaders may be variable. Our study expands this perception by showing that plant invasions can increase, decrease, or have no effect on native plants and that invasion impacts are a continuum across the invaded range. In addition, we quantified the relative contributions of recipient communities (71.39%), invasive species (21.46%) and climate (7.15%) to invasion impacts on native plant species, and analyzed how the contributions of these determinants were further explained by corresponding measured variables.

## Additional Information

**How to cite this article**: Dong, L.-J. *et al.* What determines positive, neutral, and negative impacts of *Solidago canadensis* invasion on native plant species richness? *Sci. Rep.* **5**, 16804; doi: 10.1038/srep16804 (2015).

## Supplementary Material

Supplementary Information

## Figures and Tables

**Table 1:** Contribution (%) of each category and latent variable (LV) to global explained observed variability (*R*^*2*^ = 56.78%) of relative impact index (RII).

Category	LV explaining RII	Path coefficients	Correlation	Contribution to global R^2^ (%)
Climate	MAT	−0.33***	−0.01	0.58%
	MAP	0.29**	0.13	6.58%
				7.15%
Recipient	Plant community	−0.59***	−0.64	65.88%
community	Soil abiotic properties	−0.07	−0.16	1.95%
	Soil microorganisms	0.12*	0.17	3.56%
				71.39%
Invader	*Solidago canadensis*	−0.30***	−0.41	21.46%

^*^P < 0.05; **P < 0.01; ***P < 0.001. The “path coefficient” expresses how much a response variable changes in response to changes in an explanatory variable while controlling for the effects of other explanatory variables. Therefore, a direct effect corresponds to the standardized partial regression coefficient in a multiple regression. The goodnesss-of-fit index of the path model was 0.51. The path coefficient was estimated by bootstapping.

**Table 2:** Weight of each manifest variable for its latent variable and correlations between manifest variables and relative impact index (RII) in the partial least squares path modeling.

Latent variable	Manifest variable	Weight	Correlation with RII
Temperature	MAT	1	−0.0098
Precipitation	MAP	1	0.13
Native plant community	Plant species richness	1	−0.64
Soil abiotic properties	TN	0.33	−0.072
	OC	0.17	0.014
	Silt%	0.39	−0.20
	Sand%	−0.38	0.17
Soil microorganisms	Fungal PLFAs	0.58	0.24
	Total PLFAs	0.52	0.059
	F/B ratio	−0.14	−0.027
*Solidago canadensis*	Cover	0.58	−0.34
	Density	0.57	−0.37

Weight indicates manifest variable’s bootstrapped weights. Only those manifest variables retained through stepwise regression in the final model are shown.
