# Supplementary material for: What determines positive, neutral, and negative impacts of Solidago canadensis invasion on native plant species richness?
Source: Sci Rep. 2015 Nov 17;5:16804. doi: 10.1038/srep16804 (PMC4648078; doi:10.1038/srep16804)
Supplement: Supplementary Information [file srep16804-s1.doc]

**Supplementary Information**

**What determines positive, neutral, and negative impacts of *Solidago canadensis* invasion on native plant species richness?**

Li-Jia Dong^1,#^, Hong-Wei Yu^1,#^, and Wei-Ming He^1,*^

Supplementary Table S1**:** Geographic description of study locations. The values of precipitation and temperature in the table represent mean annual precipitation and mean annual temperature of each location.

| Location | Altitude (m) | Latitude | Longitude | Precipitation  (mm) | Temperature  (^o^C) | Soil types | Habitat |
| --- | --- | --- | --- | --- | --- | --- | --- |
| Lianyungang | 31 | 34°44'N | 119°20'E | 1025 | 14.9 | Sand loam | Wasteland |
| Huaian | 40 | 33°37'N | 119°04'E | 1049 | 15.1 | Silt loam | River side |
| Zhenjiang | 15 | 32°08'N | 119°33'E | 1101 | 16.2 | Silt loam | Road side |
| Changzhou | 16 | 31°49'N | 119°56'E | 1077 | 16.5 | Silt loam | Wasteland |
| Nantong | 21 | 31°49'N | 121°05'E | 1089 | 16.2 | Silt loam | Road side |
| Shanghai | 3 | 31°06'N | 121°34'E | 1112 | 17.4 | Silt | Wasteland |
| Hangzhou | 10 | 30°19'N | 120°23'E | 1324 | 17.6 | Sand loam | River side |
| Zhoushan | 4 | 30°00'N | 122°03'E | 1286 | 17.1 | Silt loam | Road side |
| Shaoxing | 44 | 29°54'N | 120°30'E | 1374 | 17.6 | Loam | Road side |
| Ningbo | 12 | 29°50'N | 121°32'E | 1394 | 17.8 | Silt | Wasteland |
| Quzhou | 70 | 28°55'N | 118°55'E | 1503 | 17.9 | Silt | Road side |
| Taizhou | 73 | 28°33'N | 121°21'E | 1654 | 18.4 | Sand loam | Road side |
| Shangrao | 74 | 28°27'N | 117°55'E | 1779 | 18.2 | Sand loam | Road side |
| Yingtan | 53 | 28°12'N | 117°00'E | 1878 | 18.9 | Sand loam | Road side |
| Jingdezhen | 39 | 29°16'N | 117°10'E | 1695 | 18.4 | Sand loam | Road side |
| Nanchang | 44 | 28°42'N | 115°52'E | 1520 | 18.6 | Sand loam | River side |
| Jiujiang | 38 | 29°44'N | 116°01'E | 1310 | 18.1 | Loam | Road side |
| Anqing | 19 | 30°28'N | 117°04'E | 1322 | 17.5 | Loam | Road side |
| Ningguo | 54 | 30°40'N | 118°59'E | 1292 | 16.4 | Sand loam | Road side |
| Hefei | 35 | 31°48'N | 117°18'E | 1031 | 16.6 | Sand loam | Road side |
| Luan | 73 | 31°44'N | 116°30'E | 1133 | 16.4 | Loam | Wasteland |
| Huainan | 43 | 32°37'N | 117°06'E | 982 | 16.8 | Sand loam | Road side |

Supplementary Table S2**:** Minimum, maximum and average values of all the explanatory variables. Sc: *Solidago canadensis.*

|  | Minimum | Maximum | Average |
| --- | --- | --- | --- |
| MAT (^o^C) | 14.9 | 18.9 | 17.2 |
| MAP (mm) | 982.3 | 1878.4 | 1308.1 |
| Plant species richness | 1 | 9 | 3 |
| Shannon-Wiener index | 0 | 1.7 | 0.5 |
| Pielou evenness index | 0 | 0.3 | 0.1 |
| Simpson dominance index | 0.1 | 1 | 0.6 |
| Fungal PLFAs (nmol g^-1^) | 0.2 | 2.2 | 0.6 |
| Bacterial PLFAs (nmol g^-1^) | 5.1 | 71.1 | 25.1 |
| Total PLFAs (nmol g^-1^) | 10.3 | 98.4 | 33.4 |
| F/B ratio (%) | 1.0 | 6.4 | 2.9 |
| Soil pH | 5.9 | 8.7 | 7.8 |
| Organic C (g kg-1) | 0.4 | 62.5 | 20.3 |
| Total N (g kg-1) | 0.1 | 3.4 | 1.1 |
| Clay% | 0 | 18.8 | 10.6 |
| Silt% | 9.1 | 90.0 | 50.2 |
| Sand% | 3.3 | 90.8 | 47.1 |
| Sc cover (%) | 50 | 100 | 83.9 |
| Sc density | 12 | 112 | 46.5 |
| Sc height (cm) | 50 | 300 | 178.6 |

Supplementary Table S3**:** Selected manifest variables (MVs) for each latent variable (LV) through stepwise regression.

| LVs | Climate |  | Recipient community | |  | Invader | |
| --- | --- | --- | --- | --- | --- | --- | --- |
|  | MVs |  | MVs | *P( R_adj_^2^)* |  | MVs | *P( R_adj_^2^)* |
| Temperature | MAT |  |  |  |  |  |  |
| Precipitation | MAP |  |  |  |  |  |  |
| Native plant  community |  |  | 0.30 (Intercept)  -0.13*richness | <0.001  <0.001 |  |  |  |
|  |  |  |  | (40.20%) |  |  |  |
| Soil abiotic properties |  |  | 4.45 (Intercept)  -0.048*silt%  -0.043*sand%  -0.21*TN  0.0097*OC | <0.001  <0.001  <0.001  0.011  0.012 |  |  |  |
|  |  |  |  | (11.53%) |  |  |  |
| Soil microorganisms |  |  | 0.20 (Intercept)  0.69*fungi  -0.011*total  -0.12*F/B ratio | 0.039  <0.001  <0.001  <0.001 |  |  |  |
|  |  |  |  | (14.56%) |  |  |  |
| *S. canadensis* |  |  |  |  |  | 0.59 (Intercept)  -0.005*cover  -0.005*density | <0.001  0.014  0.0008 |
|  |  |  |  |  |  |  | (15.92%) |

For climatic factors, MAT and MAP were selected as MVs of temperature and precipitation (LVs) respectively. Compared with other plant diversity indices, plant species richness was chosen to represent native plant community, which explained 40.20% variance of RII (*P*<0.001). For soil abiotic properties, silt content, sand content, organic carbon and total nitrogen were selected due to their significant relationships with RII (11.53%, *P*<0.001). For soil microorganisms, stepwise regression showed fungal PLFAs, total PLFAs and F/B ratio explained 14.56% variance of RII (*P*<0.001, Table S2). The cover and density of *S. canadensis* had significantly negative relationships with RII and explained 15.92% variance of RII while the height was discarded. Accordingly, cover and density were treated as MVs for *S. canadensis*.

*Supplementary Table S4****:*** *Contribution (%) of each category and latent variable (LV) to global explained observed variability (R^2^ = 17%) of Solidago canadensis (Sc).*

| Category | LV explaining Sc | Path coefficients | Correlation | Contribution to global R^2^ (%) |
| --- | --- | --- | --- | --- |
| Climate | MAT  MAP | 0.29**  0.094 | 0.29  0.23 | 50.91%  12.77%  63.68% |
| Recipient community | Plant community  Soil abiotic properties  Soil microorganisms | 0.15*  0.23**  -0.20* | 0.11  0.055  -0.16 | 9.83%  7.70%  18.79% |
|  |  |  |  | 36.32% |

* *P* <0.05; ** *P* <0.01.
